# Breast cancer in women after withdrawing from an increased-risk MRI screening program

**DOI:** 10.1007/s00330-025-11718-7

**Published:** 2025-06-12

**Authors:** Suzanne L. van Winkel, Riccardo Samperna, Elizabeth A. Loehrer, Caroline A. Drukker, Kelly Sessink, Nico Karssemeijer, Ritse M. Mann

**Affiliations:** 1 Medical Imaging Department, Radboudumc: Radboud University Nijmegen Medical Centre, Nijmegen, The Netherlands; 2 Department of Clinical Genetics, Erasmus MC: Erasmus University Medical Center, Rotterdam, The Netherlands; 3 Department of Surgical Oncology, Netherlands Cancer Institute; Antoni van Leeuwenhoek (NKI), Amsterdam, The Netherlands; 4 ScreenPoint Medical BV, Nijmegen, The Netherlands; 5 Department of Radiology, Netherlands Cancer Institute; Antoni van Leeuwenhoek (NKI), Amsterdam, The Netherlands

**Keywords:** Breast cancer, MRI screening, Increased risk breast cancer screening, Screening indications

## Abstract

**Objective:**

This study evaluates breast cancer (BC) detection in women with ≥ 20% lifetime risk who discontinued annual breast MRI screening surveillance.

**Materials and methods:**

This retrospective single screening cohort (2003–2019) study included 3308 women who received at least one breast MRI. After verifying with the Netherlands Comprehensive Cancer Organization (IKNL), a sub-population of women who developed BC after discontinuation was identified. Screening indications, BC incidence rates, tumor characteristics, and reasons for MRI screening discontinuation were compared between women who continued and those who discontinued MRI screening using descriptive and inferential methods.

**Results:**

Among 3308 participants, 2647 discontinued MRI screening. After discontinuation, 58/2647 (2.2%) developed BC (43 invasive, 13 DCIS, 2 NA). Initial screening indications included: personal (26/58, 44.8%) or family history (17/58, 29.3%) of BC, BRCA1 (3/58, 5.2%), BRCA2 (1/58, 1.7%), chest irradiation (1/58, 1.7%), other mutations (2/58, 3.4%) and other (8/58, 13.8%). Of these, 27 continued mammography screening, detecting 23 BC-cases. Discontinuation was mainly physician-driven. Invasive tumor size after cessation of MRI screening tended to be larger compared to tumors detected while participating in MRI screening (median difference: 7.0 mm, 95% CI: 4.0–10.0, *p* < 0.001). Mean age at diagnosis was 58. Median time from last MRI to diagnosis was 5.9 years.

**Conclusion:**

Women with a personal or family history of BC commonly discontinued MRI screening, often influenced by referring physicians. However, after cessation of MRI screening, a substantial number of these women are still diagnosed with BC, challenging current MRI screening regulations. Although a last negative MRI appears to reduce the subsequent decade’s likelihood of BC, post-MRI discontinuation detected tumors are prognostically worse, highlighting the need for more personalized, extended screening.

**Key Points:**

***Question*** *Current regulations and duration of MRI screening do not suit the entire increased-risk population*.

***Findings*** *Early discontinuation disproportionately affects women with a personal or family history. Cancers detected after cessation of MRI screening seem larger and more often invasive*.

***Clinical relevance*** *Because certain women with a personal or family risk indication could benefit from a longer duration of MRI screening, extended, personalized screening beyond current guidelines is suggested*.

**Graphical Abstract:**

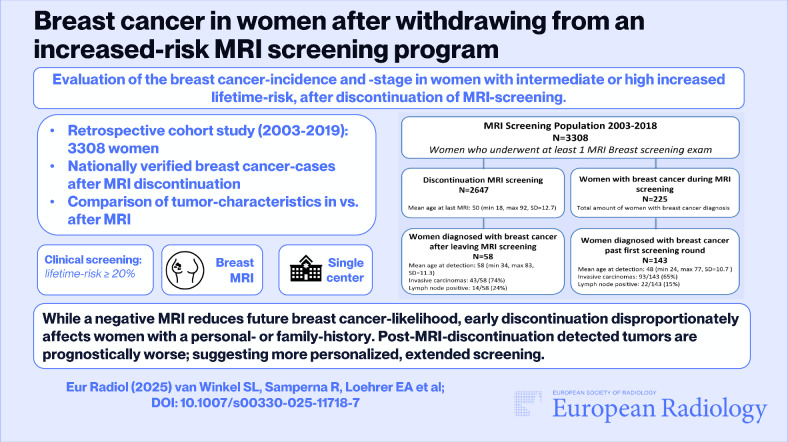

## Introduction

Breast cancer (BC) is the leading cause of cancer death in women [1]. Early detection ensures less extensive treatments and improves prognosis [1, 2]. Nationwide mammography screening programs aim for early diagnosis, achieving 20–35% BC mortality reduction amongst women aged 50–69 [3]. Mammography offers advantages: low costs, fast procedures [4], and the ability to detect microcalcifications, indicating carcinoma in situ [5]. However, mammography encounters performance variability, with limited sensitivity in dense breasts, leading to obscured lesions due to tissue superimposition [6–8]. Regrettably, 2D-mammography screening misses one in three BC cases and is less successful in women at increased risk [4–6].

Magnetic resonance imaging (MRI) is considered the most sensitive method for early BC detection [6]. Regarding specificity, MRI and mammography align [6–8]. MRI detects BC earlier, and combined MRI and mammography screening correlates with enhanced survival rates [9, 10]. Based on known risk factors (various genetic mutations, family history, and personal risk factors), the American Cancer Society guidelines stratified three risk categories, guiding which women would benefit from annual breast MRI [4, 11]. High-risk women (> 20% lifetime BC risk), including mutation carriers (BRCA1/2, PTEN, TP53, STK11, CDH1, PALB2), and those with chest irradiation before 40, should be offered annual MRI [4, 11–13]. MRI is also advised for women at intermediate-risk (15–20% lifetime risk) due to family history. However, global adherence is limited, primarily due to costs and MRI availability [14]. Average-risk women (lifetime risk < 15%) are not advised for MRI screening. However, recent recommendations do advise MRI in women with extremely dense breasts [11, 15–17]. In the Netherlands, alongside the population-based program, clinical screening in hospitals is limited to women with a lifetime BC risk exceeding 20% (considered intermediate or high risk, with high risk defined as > 50%). Women with known gene mutations or a very high lifetime risk of > 50%, based upon questionnaires (e.g., CanRisk, Tyrer-Cuzick [15, 18, 19], are offered annual MRI (age 25 or 30–60, depending on the specific indication), followed by annual mammography from age 60. For women at intermediate-risk, based on family history, prior BC and specific mutations (CHEK2, ATM, NF1 or BARD1), Dutch recommendations limit MRI screening to clinical trials [20], annual mammography starts between ages 30 and 40, depending on specific indication and risk. However, uptake in intermediate-risk women for MRI is rising, supported by compelling evidence of its benefits [11, 16, 20–25] A remarkable proportion of our hospital’s MRI screening population involves women with an intermediate-risk screening indication, for whom MRI-eligibility partially depends on patients’ and/or physicians’ preferences. MRI-indicated women usually receive complementary or alternating mammography (combined screening).

Benefits of MRI screening in women with an increased lifetime risk are clear. The criteria for discontinuing MRI screening, such as age, remain uncertain. Partly due to limited evidence on MRI screening’s effectiveness in addition to mammography for older women [4, 26]. Recommendations rely on cost-effectiveness evaluations, modeling the impact of MRI screening considering reduced tumor growth speed and decreasing breast density with age [26, 27]. Still, increased BC frequency at older age [27, 28] may render cost-effectiveness even for older women. Generally, recommendations are to discontinue MRI screening around age 60–65 and revert to standard mammography. The appropriateness of this advice varies based on individual factors like lifetime risk, tumor biology (prior BC) and breast density. Physician or patient preferences may lead to early revert to population-based mammography screening. The BC frequency in intermediate-risk women after MRI screening discontinuation is unknown. Whether satisfactory early detection is achieved after discontinuation is unclear. This study aims to evaluate the BC incidence and stage in women with intermediate or high increased lifetime risk, after discontinuation of MRI screening.

## Materials and methods

### Study design

This retrospective observational study, conducted in a consecutive single cohort of women screened with breast MRI from January 1, 2003, until December 31, 2019, within our hospital (Radboudumc: Radboud University Nijmegen Medical Center), was in line with the national privacy protecting guidelines and registered by the ethical committee (CMO registration number 2020-6325). Ethical approval was waived (Non-WMO (Dutch law on Medical-scientific Research with Humans)-research).

### Study population

**Fig. 1:**
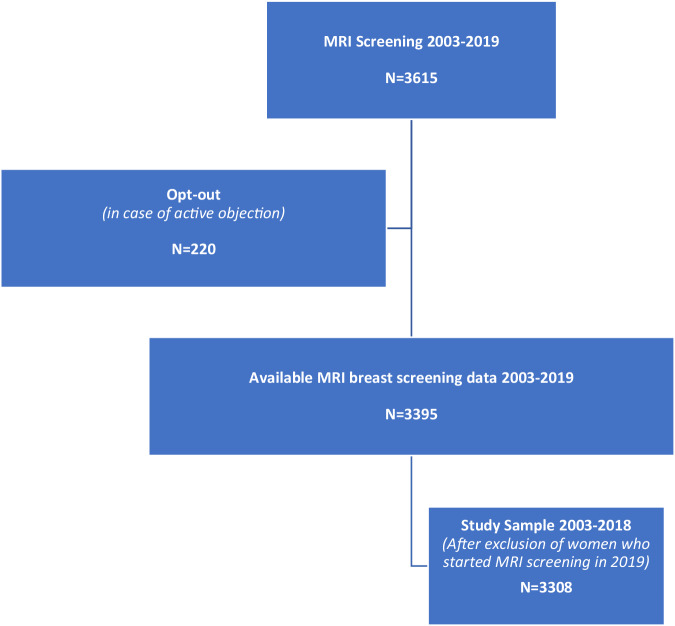
Flowchart representing the pathway from the study population, consisting of our hospital’s MRI screening population between 2003 and 2019, to the selected cases in the definitive study sample. *N*, number of included women

To facilitate verification of the return for follow-up breast MRI screening examinations, we limited our population to women who underwent at least one MRI for screening purposes until December 31, 2018. Both women screened by MRI only and women involved in combined screening with MRI and 2D-mammography were included. Since screening is annual in this cohort, we assumed women discontinued MRI screening if there was at least 13.5 months follow-up without subsequent MRI. Women who underwent MRI screening after November 15th, 2018, were considered to have remained in MRI screening (< 13.5 months of follow-up) (Fig. 1).

### Case collection

Breast screening with MRI and absence of an active objection in the electronic patient record (EPR) against anonymous data reuse for research were the only conditions for inclusion. Automated queries were designed to collect structured EPR data: screening indications, number of screenings, age and biopsy outcomes were retrieved, supplemented with manual data extraction from free text when required (e.g., unclear screening indications).

### Breast density

For women who discontinued MRI screening with available complementary or alternating mammograms, the last known volumetric density scores were obtained from mammograms using Volpara software (version 1.5.4.0). Results per image were transposed into Breast Imaging Reporting and Data System (BIRADS) V5 lexicon categories [29], averaging the per breast mediolateral oblique and craniocaudal view density levels, applying the thresholds: < 3.5% (A), < 7.5% (B), < 15.5% (C) and ≥ 15.5% (D). The highest per breast density category was assigned as the overall breast density.

### Reference standard

To ensure that BC detected in other hospitals was also captured, the Netherlands Comprehensive Cancer Organization (IKNL) was queried for follow-up information until 31-12-2020. To verify and enhance pathological information, women were also matched with the nationwide network and registry of histo- and cytopathology in the Netherlands (PALGA) [30], and relevant data were extracted. For BC diagnoses postdating the latest recorded MRI-examination, the EPR was manually consulted to determine whether: the diagnosis resulted from the latest screening, emerged as an interval cancer (detected due to symptoms within a year after the last screening or before the next scheduled screening) without intent to discontinue MRI screening, coincided with a preventive mastectomy (planned risk-reducing mastectomy) that was already planned at the last screening, or occurred after MRI screening termination (irrespective of subsequent clinical 2D-mammography). If a cancer diagnosis was made due to work-up of screening findings, resulting in a diagnosis, the cancer was regarded as screen-detected. In women who underwent mammography screening at our hospital after stopping MRI screening, it was noted whether subsequently detected cancers were mammography screen-detected or interval cancers.

### Statistical analysis

Descriptive analysis was performed to determine: the total number of women who discontinued MRI screening after initial start between 2003 and 2019, and the number of women with a malignant diagnosis during and/or after screening participation. For all women in the screening program and the women who discontinued MRI screening, the screening indications and the number of screening rounds were evaluated and compared. BC incidence in women discontinuing MRI screening was calculated and stratified by screening indication. To account for variable follow-up time after leaving MRI screening, the incident rate (IR) was expressed by cases/1000 person-years. The follow-up time was considered the period between the last known screening MRI and the last date of this study’s follow-up. In case of subsequent BC, women were censored at the date of diagnosis. For women who developed BC after discontinuing MRI screening, the following items were mapped: reason for discontinuation, whether followed by 2D-mammography screening, and if post-MRI discontinuation tumors were interval cancers. For women who developed BC after MRI discontinuation, the last known density scores were descriptively analyzed and compared to the total group of women who discontinued MRI screening. Tumor characteristics were compared between cancers detected during and after MRI screening, including tumor type, tumor size, and presence of positive lymph nodes. BCs diagnosed during the first screening round were excluded from tumor-characteristic analysis, due to its prevalence nature, where cancers tend to be more advanced. Group differences were studied using inferential statistical methods. To further analyze the tumor size differences, after performing the Shapiro-Wilkinson test for normality, the Mann-Whitney U test was performed, and the Hodges–Lehmann Estimator was calculated to determine the median difference. The Fisher exact test was used to analyze the difference in the number of lymph node-positive cases. *p*-values and 95% confidence intervals were calculated. Results were considered significant if *p* ≤ 0.05. Analysis was performed in R-studio (version 4.0.2, www.r-project.org).

## Results

### Screening participants

**Fig. 2:**
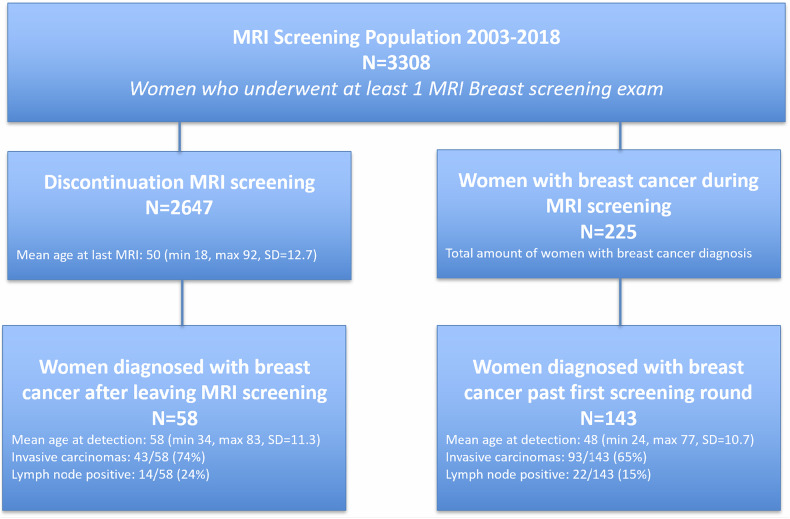
Occurrence of breast cancer diagnosis during versus after clinical MRI breast screening participation, aimed at women with an increased lifetime risk. *N*, number of included women. Due to the natural dynamics of a continuously fluctuating screening population and the duration of the selected cohort, women who discontinued MRI screening and those diagnosed with breast cancer during screening are not mutually exclusive groups. There may be overlap between these groups, and some women have (not yet) discontinued screening or been diagnosed, which is not taken into account since this does not indicate their future screening status or the length of their participation

Between 2003 and 2019, a total of 3308 women participated in MRI breast screening, on average undergoing 4 screening rounds (median 3; range 1–18; SD 3.4; 12,995 total). Of these, 2647 women ended MRI screening participation. Mean age at last MRI was 50 (median: 50; range 18–92; SD = 12.7). Mean follow-up after discontinuation was 10.3 years (median 11.7; range 2.1–17.9; SD = 4.3) (Fig. 2, Table 1). The largest populations undergoing MRI screening either had a personal or family history of BC, with smaller fractions of women being scanned for BRCA1 or BRCA2 mutations. MRI screening was more frequent discontinued in women with a personal BC history (815/899, 91%), a positive family history (669/772, 87%), and those in the ‘Other’ increased-risk subgroup (397/455, 87%), compared to women with hereditary gene mutations (BRCA1 or BRCA2: 727/1082, 67%; other genetic risk mutations: 21/43, 49%) or a history of chest irradiation (18/57, 32%) (Table 1).

**Table 1:** Baseline characteristics and descriptive analysis of the included research population

		Total	Screening indication					
			BRCA	PHBC	Family	Other	OM	ChIr
*N* (%)		3308 (-)	1082 (32.7)	899 (27.2)	772 (23.3)	455 (13.8)	43 (1.3)	57 (1.7)
Age at start of MRI screening								
	Mean (SD)	45 (12.34)	39 (11.17)	52 (10.98)	43 (10.09)	45 (11.7)	38 (11.97)	45 (11.7)
	Median (range)	45 (15–91)	37 (23–75)	53 (22–91)	43 (19–73)	45 (15–80)	38 (17–69)	37 (24–69)
Participated screening rounds								
	Total	12995	4936	3351	3078	1359	152	119
	Mean (SD)	4 (3.4)	5 (3.7)	4 (3.7)	4 (3.9)	3 (2.8)	4 (2.2)	2 (1.27)
	Median (range)	3 (1–18)	3 (1–18)	3 (1–16)	3 (1–18)	2 (1–17)	3 (1–11)	2 (1–5)
Diagnosis during MRI screening (*N* (%))		225 (-)	127 (56.4)	40 (17.8)	35 (15.6)	16 (7.1)	4 (1.8)	3 (1.3)
Cancer detection rate (‰)		17.3	25.7	11.9	11.4	11.8	26.3	25.2
Diagnosis during MRI minus first round (*N* (%))		143 (-)	76 (53.4)	24 (16.8)	27 (18.9)	12 (8.4)	3 (2.1)	1 (0.7)
Age 1st diagnosis during screening								
	Mean (SD)	48 (10.7)	45 (9.7)	56 (11.3)	48 (8.3)	53 (10.5)	40 (9.9)	42 (3.2)
	Median (range)	47 (24–77)	44 (27–59)	55 (30–77)	47 (35–76)	53 (37–70)	39 (31–53)	41 (40–46)
Age 1st BC diagnosis during screening past first round								
	Mean (SD)	48 (10.2)	45 (8.7)	56 (11.9)	48 (8.0)	52 (11.0)	43 (9.5)	46 (-)
	Median (range)	48 (27–77)	43 (27–59)	55 (30–77)	47 (37–76)	53 (37–70)	43 (34–53)	46 (-)
Women leaving screening (*N* (%))		2647 (-)	727 (27.5)	815 (30.8)	669 (25.3)	397 (15.0)	21 (0.8)	18 (0.7)
Age last MRI screening (in case of discontinuation)								
	Mean (SD)	50 (12.7)	44 (12.1)	57 (11.2)	47 (10.9)	52 (11.9)	41 (14.9)	46 (12.5)
	Median (range)	50 (18–92)	42 (24–78)	57 (23–91)	47 (20–79)	52 (18–81)	38 (18–69)	43 (26–69)
Breast density category (in case of discontinuation)								
	A	59	38	7	8	5	1	0
	B	328	175	82	49	13	5	4
	C	398	172	110	95	21	0	0
	D	345	128	83	113	16	3	2
	NA	1517	214	533	404	342	12	12
FU after discontinuation (years)								
	Mean (SD)	10.3 (4.3)	8.7 (4.1)	11.1 (4)	11.3 (4)	10.3 (4.4)	3.4 (1)	3.9 (1.27)
	Median (range)	11.7 (2.1–18.0)	8.3 (2.1–17.9)	11.5 (2.2–18.0)	11.9 (2.2–18.0)	10.5 (2.2–18.0)	3.2 (2.2–5.5)	3.5 (2.2–6.00

Cancer detection rate is expressed as the number of cancers detected per 1000 MRI screenings (‰)

*BC* breast cancer, *BRCA* BRCA1/BRCA2 mutation carriers, *PHB* personal history of BC, *Family* family history of BC, *OM* other risk mutations, *Other* remaining other risk indications for MRI screening, *ChIr* former chest irradiation

### Breast cancer incidence

During MRI screening, the overall cancer detection rate (CDR) was 17.3/1000; BC (252 lesions) was detected in 225/3308 (6.8%) women (mean age at detection: 48 (median: 47; range 24–77, SD = 10.7)). 91 BC lesions (82 women) were detected during the first screening round and excluded from analysis on tumor characteristics. 143 women (161 BC lesions) were diagnosed during follow-up rounds (mean age at detection: 48 (median: 48; range 27–77, SD = 10.2)) (Fig. 2, Table 1). During MRI screening, > 50% of cancers were detected in mutation carriers (CDR = 25.8/1000). The CDR was similarly high for women with a history of chest irradiation (CDR = 25.2/1000), with lower detection rates in the other populations (Table 1, CDR = 11.9/1000).

After MRI screening discontinuation, 58/2647 (2.2%) women presented with BC; 43 women with invasive BC (46 lesions) and 13 women with ductal carcinoma in situ (DCIS) (14 lesions). For two women (3 lesions), BC was reported to IKNL without available information on size and stage. One woman had both DCIS and an invasive tumor (Table 2). Of the 58 women who developed BC after discontinuing MRI screening, 10 had prior BC diagnosed during their MRI screening participation and were diagnosed with recurrent or second primary lesions after leaving MRI screening (11 lesions: 7/11 in situ, 4/11 invasive) (Table 3). Of the women who developed BC after discontinuation, 51/58 (88%) had a personal or family history of BC (Tables 2, 3). They underwent on average 3 screening rounds (median 3; range 1–12; SD 10.8; 196 total), with the mean age at subsequent diagnosis being 58 (median: 58, range: 34–83, SD = 11.3) (Table 3). BC-incidences until follow-up completion in discontinuing women were: 58/2647 [2.2%] (total), 4/727 [0.6%] (*BRCA1/2*), 2/21 [9.5%] (other risk mutations), 1/18 [0.6%] (chest irradiation), 26/815 [3.2%] (personal history), 17/669 [2.5%] (family history) and 8/379 [2.1%] (other).

**Table 2:** Descriptive analysis of tumor characteristics for the tumors that were diagnosed during the MRI screening period versus diagnoses that occurred after discontinuation of MRI screening, total and stratified on screening indication

	Total			Screening indication																	
				BRCA			PHBC			Family			Other			OM			ChIr		
	DS	DS-1	AS	DS	DS-1	AS	DS	DS-1	AS	DS	DS-1	AS	DS	DS-1	AS	DS	DS-1	AS	DS	DS-1	AS
*N* cases [lesions] (% cases)	225 [252] (-)	143 [161] (-)	58 [63] (-)	127 [141] (56.4%)	76 [85] (53.1%)	4 [4] (6.9%)	40 [41] (17.8%)	24 [25] (16.7%)	26 [26] (48.1%)	35 [42] (15.6%)	27 [31] (18.9%)	17 [19] (29.3%)	16 [19] (7.1%)	12 [15] (8.4%)	8 [9] (13.8%)	4 [5] (1.7%)	3 [3] (2.1%)	2 [1] (3.4%)	3 [4] (1.3%)	1 [2] (0.7%)	1 [2] (1.7%)
Invasive																					
Cases [lesions] (*N*)	145 [151]	93 [98]	43 [46]	86 [90]	52 [55]	3 [3]	25 [25]	17 [17]	20 / 20	19 [19]	12 [12]	14 [16]	11 [13]	9 [11]	5 [6]	3 [3]	2 [2]	0	1 [1]	1 [1]	1 [1]
Size [mm] (mean (SD))	12.7	12.1	20.7	10.9	10.2	16.7	14.8	11.1	21.3	12.7	11.1	21.9	22.8	24.3	19.5	7.3	10.5	NA	9	9	13
Size [mm] (median (range))	10 (< 1–70)	9.0 (< 1–65.0)	16.0 (1.0–74.0)	9 (< 1–46)	< 1 (< 1–46.0)	16.0 (14–16.7)	12 (< 1–70)	10.5 (< 1–29)	15.5 (2.0–52.3)	9 (2–35)	7 (2–35)	17.0 (1.0–74.0)	23 (6–65)	23 (7–65)	15.0 (9.0–49.0)	5 (1–16)	10.5 (5–16)	NA	9 (-)	9 (-)	13 (-)
Size = NA (lesions)	4	2	3	2	1	0	2	1	2	0	0	1	0	0	0	0	0	NA	0	0	0
Lymph node positive (cases (*N*))	36	22	14	20	12	0	5	3	8	6	2	4	5	5	2	0	0	0	0	0	0
In situ																					
Cases [lesions] (*N*)	80 [87]	46 [50]	13 [14]	42 [48]	24 [28]	1 [1]	13 [13]	5 [5]	5 [5]	16 [17]	13 [13]	2 [2]	4 [4]	2 [2]	3 [3]	2 [2]	1 [1]	1 [2]	3 [3]	1 [1]	1 [1]
Size [mm] (mean (SD))	14.8	12.2	22.0	10.1	8.6	NA	NA	NA	17.0	30	30	NA	22.8	24.3	19.5	50	NA	41	8	NA	22
Size [mm] (median (range))	8 (< 1–50.0)	6.0 (< 1–50.0)	19.5 (3.0–41.0)	6 (< 1–34)	3 (< 1–34)	NA	NA	NA	15.0 (15.0–21.0)	30 (10–50)	30 (10–50)	NA	23 (6–65)	23 (7–65)	15.0 (9.0–49.0)	50 (50–50)	NA	41 (41–41)	8 (3–13)	NA	22 (-)
pTX	3	3	0	1	0	0	1	1	0	1	1	0	0	0	0	0	0	0	0	0	0
pTNA	11	10	3	2	0	0	2	2	1	5	5	1	2	2	0	0	0	0	0	0	0
Size = NA (IS/pTNA/pTX) (lesions)	82	48	9	39	19	1	15	7	3	20	16	3	6	4	1	1	1	0	1	1	0

*DS* diagnosed during MRI screening, *DS-1* diagnosed during screening after exclusion of the diagnoses during the first round, *AS* diagnosed after discontinuation of MRI screening, *BC* breast cancer, *BRCA* BRCA1/BRCA2 mutation carriers, *OM* other risk mutations, *PHB* personal history of BC, *Family* family history of BC, *Other* remaining other risk indications for MRI screening, *ChIr* former chest irradiation, *pT* pathological TNM stage, *pTX* undefined/primary tumor cannot be assessed, *NA* not applicable/not applicable due to missing information

**Table 3:** Women with a BC diagnosis after MRI screening discontinuation: descriptive analysis for the total number of cases/lesions and stratified on MRI screening indication

Women with BC diagnosis after discontinuation of MRI screening	Screening indication	BRCA	OM	ChIr	PHBC	Family	Other	Total
# Cases (*N*)		4	2	1	26	17	8	58
Age at start MRI screening	Mean	42	39	41	52	48	51	50
	Median	37	39	41	55	45	50	58
	Range	29–65	35–42	41	28–70	32–72	45–58	28–72
	SD	16.3	4.9	NA	10.9	11.1	4.7	10.8
Participated screening rounds (*n*)	Mean	2	2	2	4	4	2	3
	Median	2	2	2	2	3	1.5	3
	Range	1–4	1–3	2–2	1–9	1–12	1–5	1–12
	SD	1.5	1.4	NA	2.9	3.5	1.4	2.8
	Total	9	4	2	95	70	16	196
Former diagnosis during MRI screening	Invasive BC (Cases/lesions)	2 (2)	0 (0)	0 (0)	0 (0)	2 (2)	0 (0)	4 (4)
	In situ BC (Cases/lesions)	0 (0)	0 (0)	0 (0)	2 (2)	4 (4)	1 (1)	7 (7)
	Total (cases/lesions)	2 (2)	0 (0)	0 (0)	2 (2)	5 (6)	1 (1)	10 (11)
Age at BC diagnosis after discontinuation	Mean	49	43	44	61	57	61	58
	Median	45	43	44	61	54	61	58
	Range	34–72	38–47	44	38–83	43–81	53–69	34–83
	SD	17.8	6.4	NA	11	10.6	5.4	11.3
Time interval last MRI BC diagnosis after discontinuation	Mean	4.5	3.4	1.8	6.2	6	9	6.2
	Median	4	3.4	1.8	6.4	6.1	8.9	5.9
	Range	2.4–7.6	2.8–4.1	1.8	1.2–14.9	1.7–13.9	4.6–13.8	1.2–14.9
	SD	2.2	0.9	NA	3.5	3.6	3.5	3.6
Breast density at last screening exam (*N*)	A	1	0	0	1	0	0	2
	B	1	2	0	10	6	1	20
	C	0	0	1	10	5	4	20
	D	2	0	0	4	6	4	16

*BC* breast cancer, *BRCA* BRCA1/BRCA2 mutation carriers, *OM* other risk mutations, *PHB* personal history of BC, *Family* family history of BC, *Other* remaining other risk indications for MRI screening, *ChIr* former chest irradiation, *NA* not applicable/not applicable due to missing information

### Adjusted incidence rates per screening-indication

After adjustment for variable follow-up person-time, IR were: 58/26,916.44 person-years [2.15/1000, CI: 2.13–2.17] (total), 4/6450.11 person-years [0.62/1000, CI: 0.61–0.64] (BRCA1/2), 2/67.97 person-years [29.43/1000, CI: 28.14–30.72] (other risk mutations), 1/68.90 person-years [14.51/1000, CI: 13.61–15.41] (chest irradiation), 26/8923.17 person-years [2.91/1000, CI: 2.87–2.95] (personal history), 17/7464.49 person-years [2.28/1000, CI: 2.25–2.31] (family history) and 8/3941.80 person-years [2.03/1000, CI: 1.99–2.07] (other). For women with the most common discharged screening indications (personal/family history of BC and other), IR was 51/20,329.46 person-years [2.51/1000, CI: 2.49–2.53].

### Reasons for MRI screening discontinuation

Reasons for MRI discontinuation in women with subsequent BC were: age > 60 (4/58, 6.9%), no indication according to referring physician (29/58, 50.0%), personal wish (2/58, 3.4%), unknown (20/58, 34.5%) and medical contra-indication (3/58, 5.2%).

### Breast density

For 23/58 women diagnosed after MRI screening discontinuation, density scores were available based on their last mammogram. However, for the remaining 35 women, no mammographic density scores could be retrieved: screening mammograms were unavailable (31/35) or Volpara could not process the available mammograms (4/35). Only for these 35 women, we were able to supplement the density scores based on their last screening MRI. The last known density scores were as follows: 2/58 A (3%), 20/58 B (34%), 20/58 C (34%), 16/58 D (28%) (Table 3). Among all women who discontinued MRI screening, 1130 women had density scores available: 59/1130 (5.2%) A, 328/1130 (29.0%) B, 398/1130 (35.2%) C and 345/1130 (30.1%) D (Table 1). There was no statistically significant difference in breast density distributions between the compared groups (chi-square = 1.06; *p*-value = 0.79).

### Detection methods in subsequent diagnosis

Of women with subsequent BC, 27/58 continued 2D-mammography screening in our hospital: 23/27 were diagnosed screen-detected, and 4/27 women presented with interval cancer. Due to age, the 31 remaining women were discharged to population-based biennial (2D-mammography) screening. Five of these women were diagnosed after recall from population-based screening. The detection method in the remaining 26 women is unknown. The median time interval between the last screening MRI and a subsequent diagnosis was 5.9 years (mean 6.2; range 1.2–14.9; SD 3.6) (Table 3).

### Histological tumor characteristics

50/161 (31%) cancers detected in subsequent MRI screening rounds were DCIS (Table 2). Of the lesions detected after discontinuing MRI screening, 14/64 (22%) involved DCIS. The median size of invasive cancers detected post-MRI discontinuation was 16.0 mm (mean: 20.7; range: 1.0–74.0 mm), compared to a median size of 9.0 mm (mean: 12.1; range: < 1–65.0) for invasive tumors detected during MRI screening participation (Table 2). The Hodges–Lehmann estimate of the median difference was 7.0 mm (95% CI: 4.0–10.0, *p* < 0.001). 14/43 women (33%) with invasive BC detected after discontinuation had positive lymph nodes, compared to 22/93 women (24%) with invasive BC that was detected while still in MRI screening (Fig. 2, Table 2). In case of invasive BC, women diagnosed after discontinuing MRI screening had 1.56 (95% CI 0.64–3.69; *p* = 0.30) times the odds of positive lymph nodes at detection compared to those diagnosed while still participating.

## Discussion and conclusion

This study assessed BC incidence in women initially enrolled in an increased-risk MRI screening program who later discontinued participation. At an average last MRI age of 50, and after a mean 10.3-year follow-up, 58/2647 (2.2%) women had post-MRI discontinuation detected BC. In the Netherlands, women’s average 10-year BC risk at age 50 is estimated at 3.6% [1]. A negative last MRI suggests a reduced likelihood of BC diagnosis in the next decade, especially considering the higher risk of study participants. However, given the varying 10-year BC risk by age, our population’s age range of 18–92 may limit comparability. After adjusting for the highly variable follow-up time per person, the indication-stratified average IR of subsequent BC was substantially higher in women with ‘other risk mutations’ and chest irradiation. Although this is to be expected due to the indication associated lifetime risks, these findings should be interpreted with caution, as the absolute numbers in these subgroups—particularly among women who discontinued MRI screening—are very low, limiting the reliability of conclusions. The IR of women with screening indications that were most often discharged (personal-/family history of BC and other) was 51/20,329.46 person-years [2.51/1000, CI: 2.49–2.53], which is 4 times the IR of (re)occurrence of BC after discontinuation of MRI screening of woman with BRCA1/BRCA2 mutations (4/6450.11 person-years [0.62/1000, CI: 0.61–0.64]). This suggests that the current protocol serves BRCA1/BRCA2 women well; at the same time, there is room for improvement regarding more personalized screening for women with other indications. We do note that limited information was available on competing events, such as prophylactic mastectomies or death. This is most relevant in BRCA1/BRCA2 women, often withdrawing from MRI screening after undergoing prophylactic mastectomies. The available data on ‘date of death’ proved to be insufficiently complete and reliable; we were unable to compensate for this by national verification. The average time to BC diagnosis after MRI screening discontinuation is at 5.8 years, which is quite long, which also points in the direction of a reduced likelihood of a BC diagnosis after a last negative MRI. However, subsequent BC’s were less likely to be DCIS and on average larger (7.0 mm median difference) than cancers detected within the program. The significant difference between the size of tumors detected during versus after discontinuation of MRI screening supports the assumption that these women might have benefited from longer MRI screening participation. We did not observe a significant difference in the proportion of cases with positive lymph nodes. The lack of a significant difference may be influenced by the high BC frequency in women with genetic mutations. In these women, cancers unfortunately metastasize early; consequently, positive lymph nodes are common. Previous research showed that 17% of BC in women undergoing MRI screening because of a positive family history had positive lymph nodes [31]. In our study, 28% of women with post-MRI discontinuation detected BC, and a positive family or personal history of BC had positive lymph nodes.

The absence of statistically significant differences in breast density between the groups (chi-square = 1.06, *p* = 0.79) suggests that breast density may not be a reliable factor for further stratifying MRI screening. However, the large amount of missing data, due to unavailable or unprocessed mammograms, limits the strength of this result. Among the women who were diagnosed after discontinuation, documented explanations show that neither age nor personal wish is the main explanatory factor to discontinue. The large proportion of cases in which “No indication according to referring physician” was noted suggests that the (subjective) choice of the attending physician strongly influenced whether or not the MRI screening was continued. MRI screening was most commonly discontinued in women with a personal or family history of BC, which reflects the shifting and debated MRI screening eligibility for women with intermediate-risk indications over time and may have led to premature referrals to single mammography screening. Consistent with other studies, BC detection was less frequent in these women compared to those at very high risk, such as those with hereditary germline mutations [32–34]. Dutch MRI screening recommendations for women with an increased risk based on family history have varied, mainly due to cost-effectiveness concerns [15, 35]. Maintenance of MRI screening was driven by personal choice, persistence, or the referring physician’s preference. Since these women represent the largest subgroups, this contributes to the high rate of discontinuation in our cohort due to the lack of clear guidelines for MRI screening. Additionally, a screening cohort spanning 2003–2019 naturally fluctuates due to factors such as age, health changes, transitions to population-based or clinical mammography screening, personal choice, or even death. A recent cost-effectiveness study supports familial-risk MRI screening, offering a 18 month screening-interval or alternated with annual mammography [36], Dutch guidelines are currently under revision to incorporate this approach, replacing the current annual mammography screening recommendation from age 40, followed by referral to population-based screening from age 50 [15]. Our results support lower MRI screening frequency, considering the relatively long time to subsequent BC. MRI screening in women with prior BC has never been formally indicated and is typically at the discretion of the referring physician or upon patient request. However, prior studies reveal that MRI screening in these women doubles sensitivity compared to mammography and detects a substantial number of cancers following negative mammography [14, 37, 38]. Hence, it is unsurprising that this practice remains common. Despite the improvement in early detection, MRI screening carries a higher risk of false positives and overdiagnosis, which should be carefully weighed against its benefits [38]. The median age at BC detection after discontinuation was < 60, which is the age limit in most MRI screening guidelines [15], would justify MRI screening continuation in increased-risk programs until at least that age. Despite our study involving a large series of women in an MRI screening program followed for 16 years, tracking (post-MRI discontinuation) BC occurrences, there are limitations. Cases were collected at a single Dutch academic hospital, limiting the size of subgroups. Extending this study is necessary for meaningful risk stratification. While the current observational single-cohort study design is valuable for understanding disease progression and screening efficacy, it is limited by potential (selection) bias due to uncontrolled and non-visible factors influencing decisions regarding eligibility, duration and discontinuation of MRI screening participation. Particularly among intermediate-risk women in our MRI screening population, whose indication for MRI is a subject of debate. However, the increased IR of BC (re)occurrence after MRI discontinuation compared to non-controversial high-risk MRI screening attenders remains noteworthy. This longitudinal observer study does not assess a single MRI protocol but reflects clinical practice, including the gradual increase of spatial resolution, implementation of ultrafast MRI, T2 and DWI, and adoption of shorter (abbreviated) protocols. Protocols adhered to contemporary standards and aligned with the requirements of the EUSOBI breast MRI guidelines [15, 39]. Our inability to determine detection methods in women who developed BC after discontinuing MRI screening, not followed in our hospital, implies potential underestimation of interval cancer frequency. The observed shift in reported clinical stage of BC diagnosed after MRI discontinuation implies a fraction of interval cancers, similar to what is observed in the national screening (± 25%).

In conclusion, most BCs detected after MRI screening discontinuation occurred in women with a personal or family history of BC, tending to be larger and more often invasive compared to those detected during screening. Despite a lower overall BC incidence in the first 10 years after a negative MRI, our results suggest that extended MRI screening may benefit certain women with a personal or family history of BC. As the necessity and benefits of MRI remain debated, maintaining screening at a reduced interval could support timely detection while balancing the burden and costs. Further research to refine risk stratification seems valuable.

## Abbreviations

BC: Breast cancer
DCIS: Ductal carcinoma in situ
EPR: Electronic patient record
IKNL: Netherlands Comprehensive Cancer Organization
IR: Incident rate
MRI: Magnetic resonance imaging
PALGA: Dutch Pathology Registry
SD: Standard deviation
